# Supplementary material for: Polymer-coated carbon nanotube hybrids with functional peptides for gene delivery into plant mitochondria
Source: Nat Commun. 2022 May 16;13:2417. doi: 10.1038/s41467-022-30185-y (PMC9110379; doi:10.1038/s41467-022-30185-y)
Supplement: Supplementary file 1 — Supplementary Information [file 41467_2022_30185_MOESM1_ESM.pdf]

Polymer-coated carbon nanotube hybrids with functional peptides for gene delivery into plant mitochondria

Law *et al.*

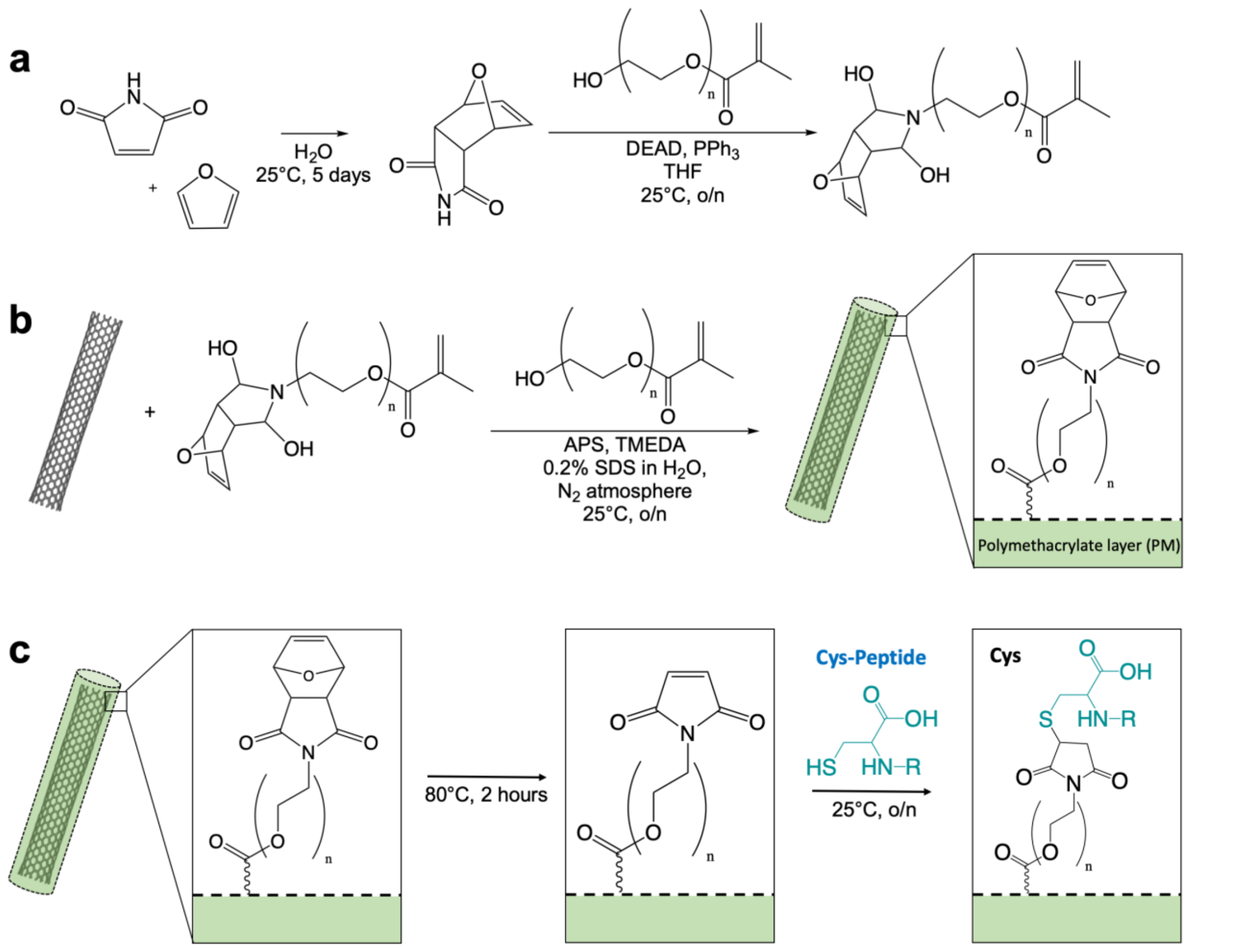

Supplementary Fig. 1. Overall schematic of the SWNT-PM-Peptide delivery system.

(a) Synthesis of the protected maleimide monomer used for coating the SWNT. **(b)** Cross-linking of the polymethacrylate maleimide layer by micelle polymerization. **(c)** Cys-containing peptides are functionalized to deprotected maleimide via Michael addition.

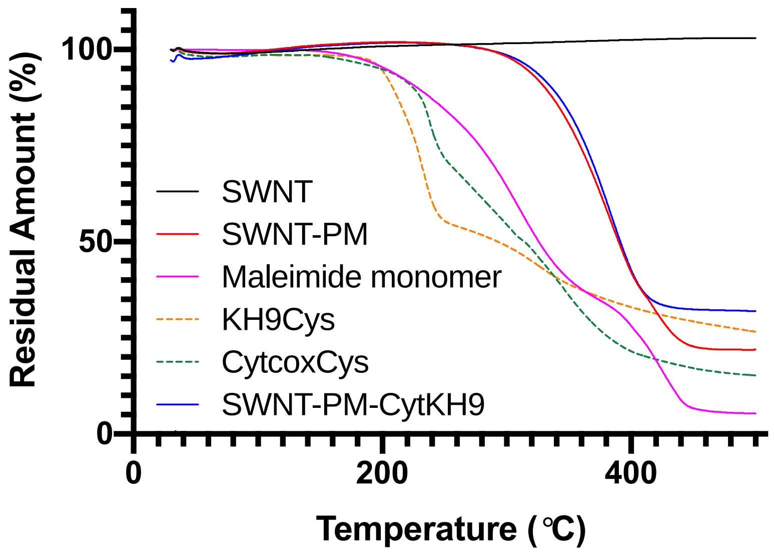

Supplementary Fig. 2. TGA analysis of the SWNT NCs.

TGA analysis curves for the SWNT NCs and unconjugated peptides showing their mass loss profile during pyrolysis. Source data are provided as a Source Data file.

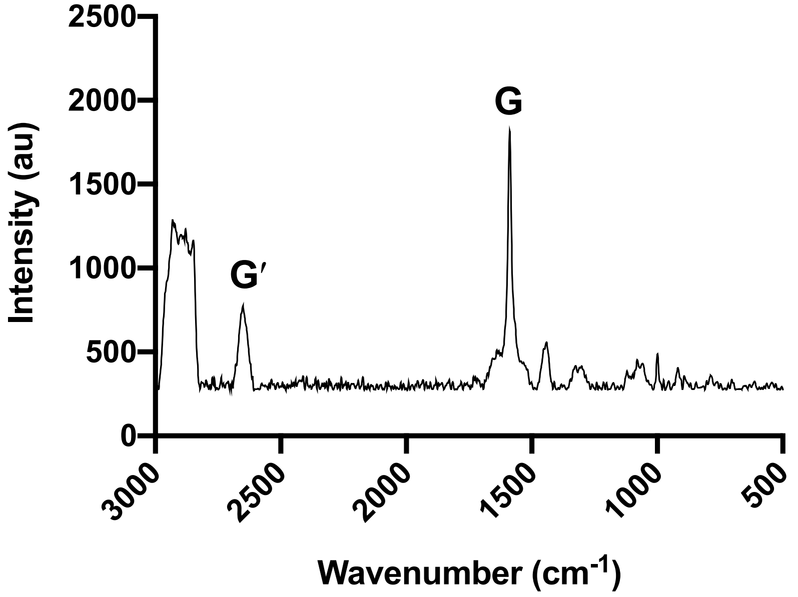

Supplementary Fig. 3. Raman spectra of SWNT-PM.

The Raman spectrum of polymethacrylate maleimide adsorbed on SWNTs retains the characteristic G and G`-bands found in SWNTs. Source data are provided as a Source Data file.

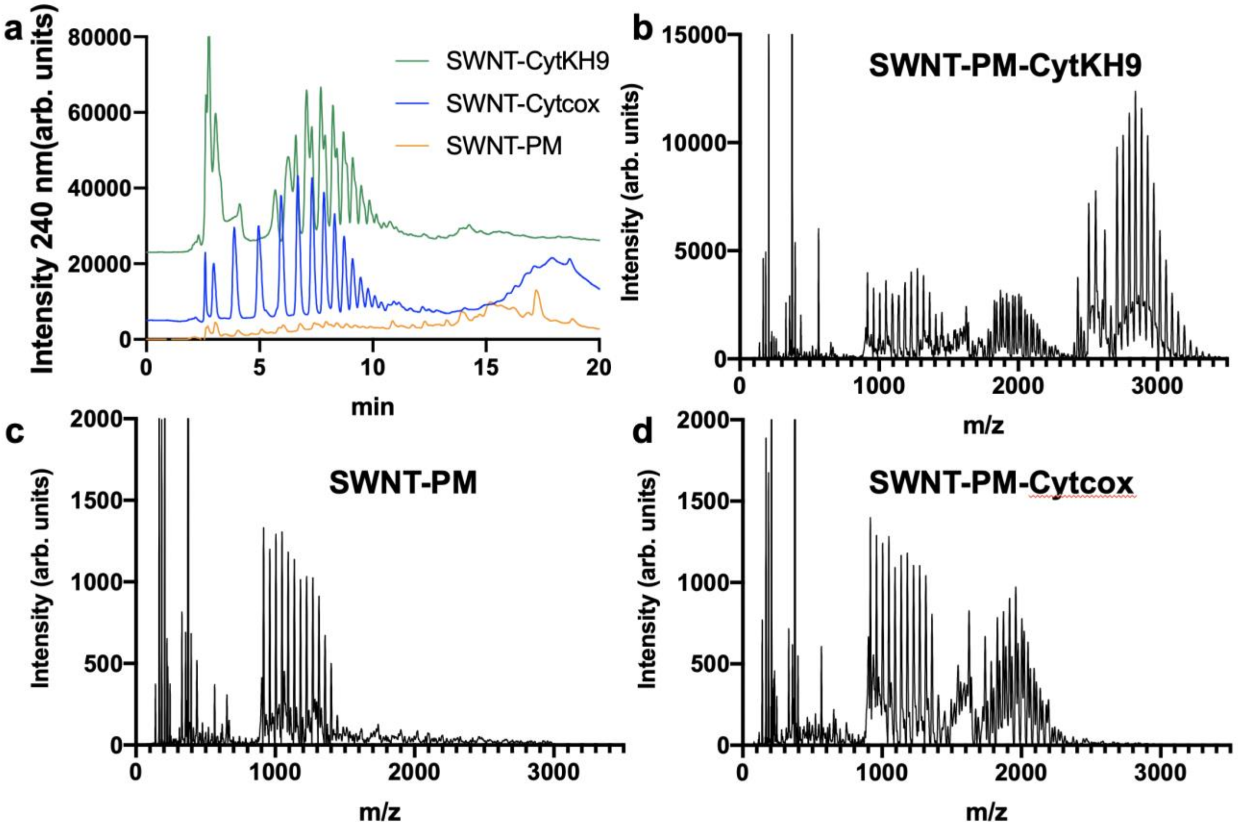

Supplementary Fig. 4. HPLC chromatograms and MALDI-TOFMS spectra of cleaved peptide fragments from SWNT-PM and SWNT-PM-Peptide.

(a) HPLC chromatograms of the methacrylate-PEG conjugated peptides cleaved from SWNT-PM (orange) and SWNT-PM-Peptide (green and blue). **(b-d)** MALDI-TOFMS spectra of cleaved peptide fragments after separation by HPLC from **(b)** SWNT-PM-CytKH9, **(c)** SWNT-PM, and **(d)** SWNT-PM-Cytcox. The single maleimide methacrylate monomer unit and unconjugated Cytcox and KH9 peptides were *m/z* = 155, *m/z* = 1602, and *m/z* = 2507, respectively. The lowest identifiable m/z peaks for the conjugated species were *m/z* = 919, *m/z* = 1757, and *m/z* = 2662 and represent a single methacrylate monomer unit conjugated to the respective peptides. MS spectra data are included in Supplementary Data 3-5. Source data are provided as a Source Data file.

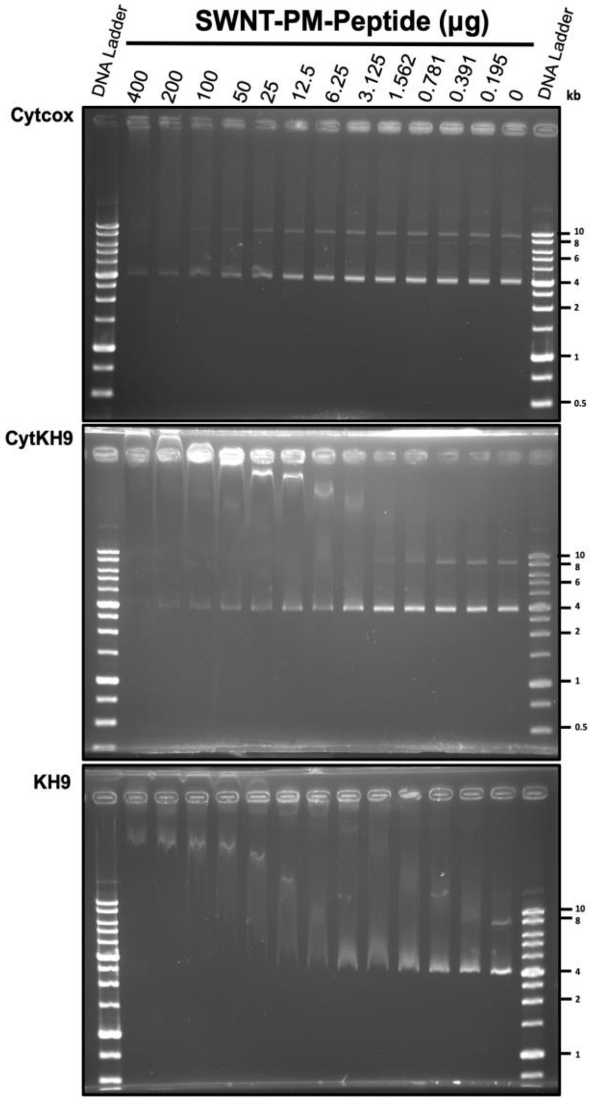

Supplementary Fig. 5. Binding of pDNA to SWNT-PM-Peptide NCs.

Representative gel shift electrophoresis mobility assay used for quantification in Fig. 2e. The main unbound band at approximately 4kb was used for comparison in the binding abilities of the SWNT-PM-Peptide NCs.

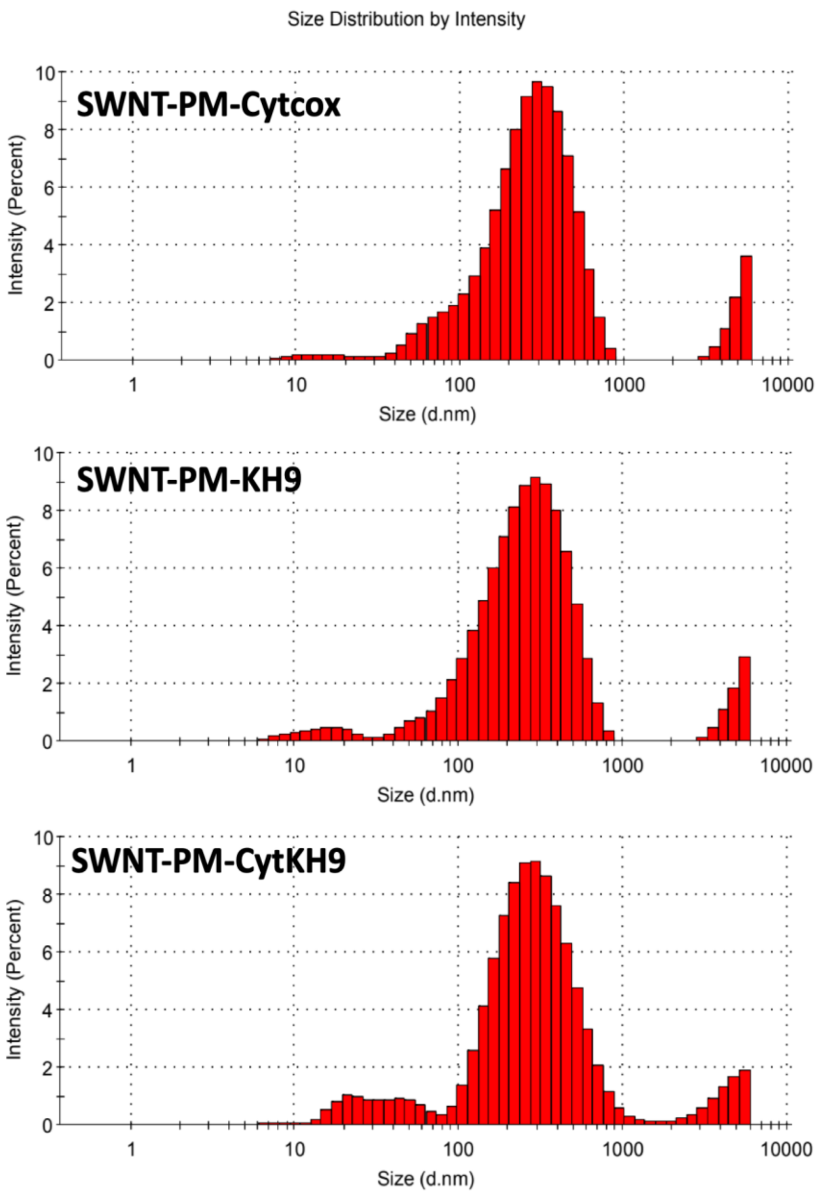

Supplementary Fig. 6. Dynamic light scattering measurements of SWNT-PM-Peptide NCs.

DLS measurements of particle size showed similar results with those observed using AFM.

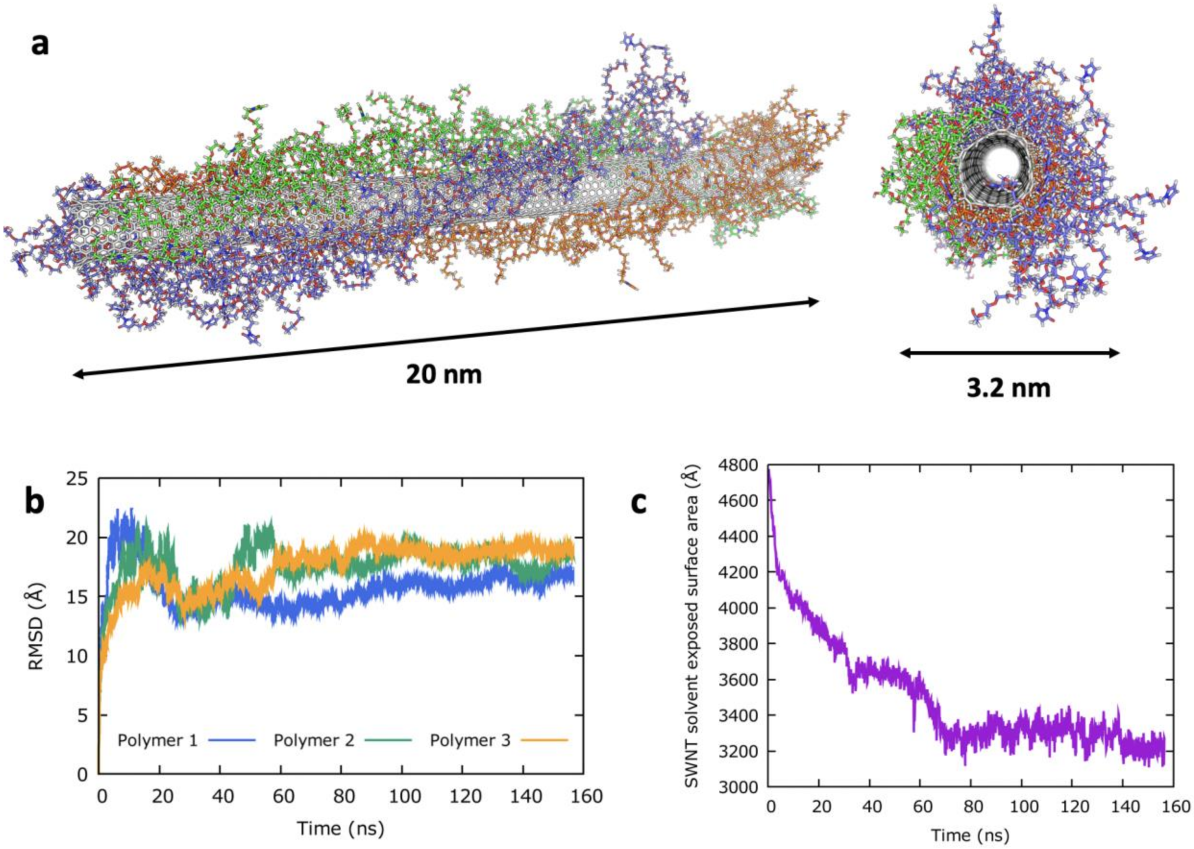

Supplementary Fig. 7. MD simulations of polymethacrylate maleimide polymer adsorption on SWNTs.

(a) Lateral and cross-sectional views of the last snapshot of an MD simulation of SWNT (20 nm) with three 50 repeating polymethacrylate maleimide unit polymer molecules (green, orange and blue) after 150 ns of equilibration time. **(b)** The RMSD of the heavy atoms of the three polymer molecules (green and blue) over the simulation time of 150 ns shows that equilibrium has been reached. **(c)** The decrease in solvent-exposed surface area of the SWNT molecule over the simulation time of 150 ns indicates efficient coverage by the polymer molecules.

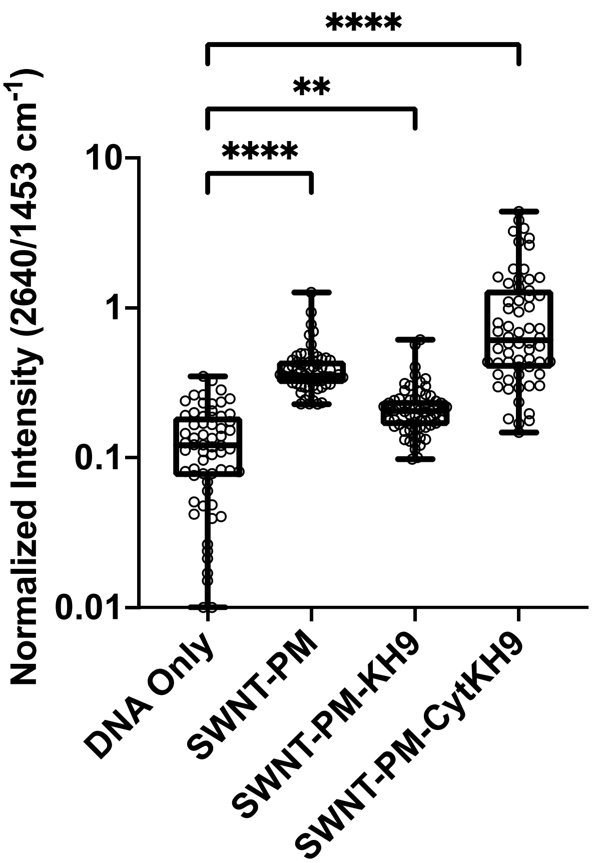

Supplementary Fig. 8. Normalized G`-band Raman intensities from isolated mitochondria. Quantified normalized G-band peak (2640 cm^{-1}) intensities from Fig. 4b for isolated mitochondria samples. Relative values from 64 individual spectra (n=64) are shown for each individual sample in their respective box and whisker plots depicting the maxima, minima, median and the first (25%) and third (75%) quartiles. Statistical significance between individual samples was determined by one-way ANOVA. *P*-values are <0.0001 for comparisons between DNA only and SWNT-PM and SWNT-PM-CytKH9, and 0.0073 for DNA only and SWNT-PM-KH9. ***P*<0.01, ****P*<0.001, *****P*<0.0001. Source data are provided as a Source Data file.

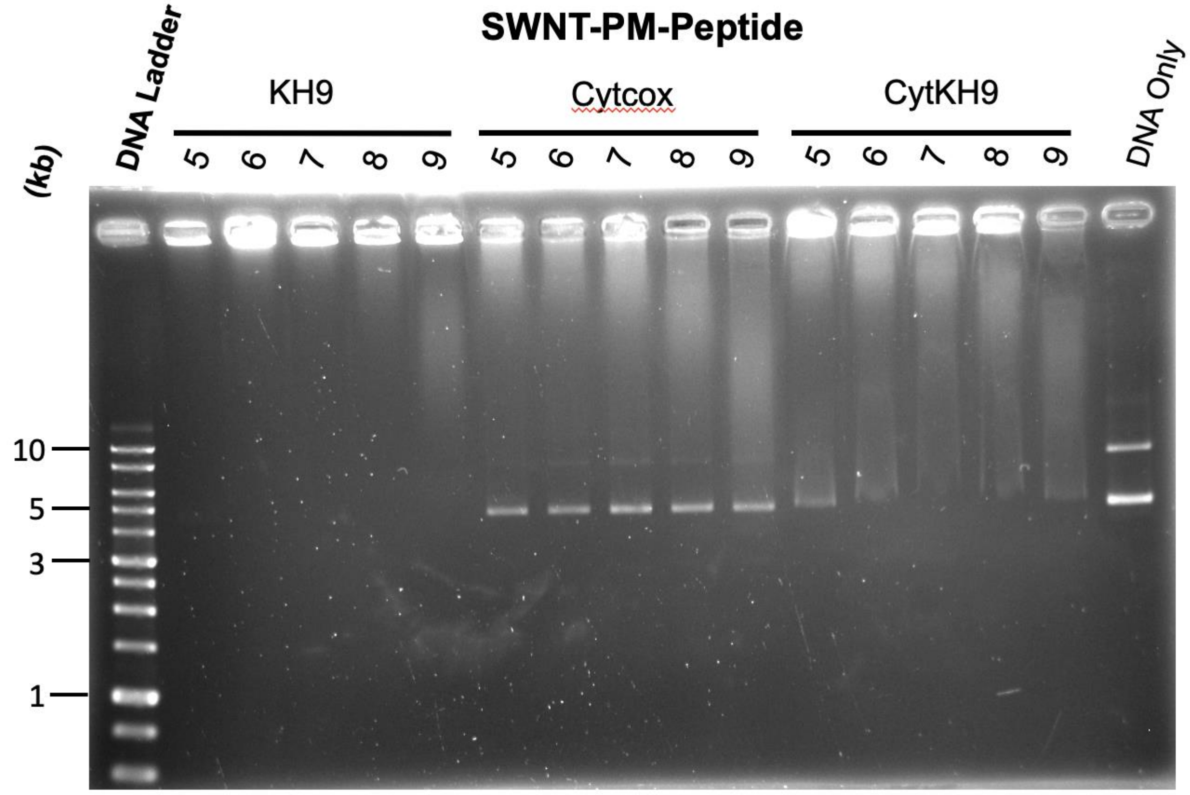

Supplementary Fig. 9. The effect of pH on SWNT-PM-Peptide/pDNA complexes.

Effect of increasing pH from 5 to 9 on the release of pDNA from the respective SWNT-PM-Peptide.

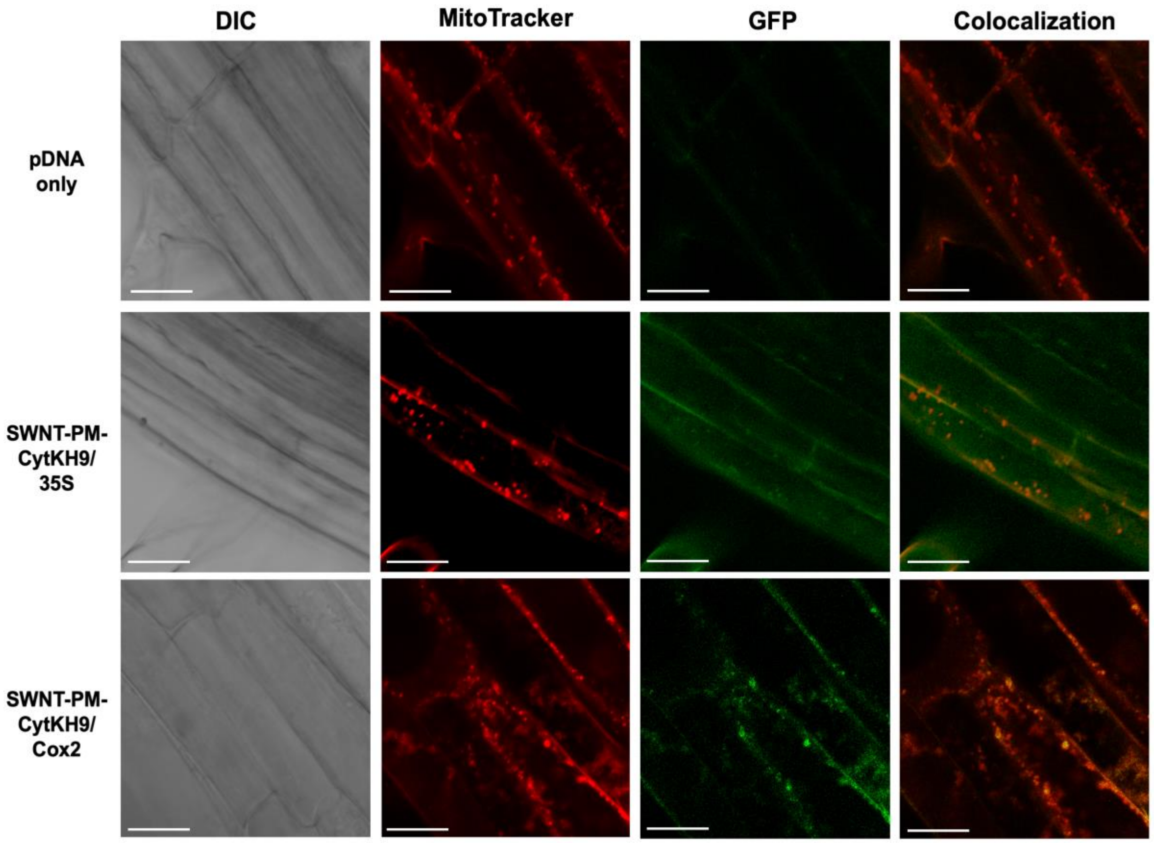

Supplementary Fig 10. Confocal laser scanning microscopy images of *A. thaliana* root cells 18 h post infiltration with SWNT-PM-CytKH9/pDNA complexes.

Higher magnification images of *A. thaliana* infiltrated with SWNT-PM-CytKH9/pDNA shown in Fig. 5b. Mitochondria were stained with MitoTracker Red (CMXRos), and colocalization analysis was performed on GFP expression and MitoTracker signals. Representative confocal laser scanning microscopy images of *A. thaliana* root cells from a minimum of 5 seedlings for each condition (n=5) 18 h post infiltration with SWNT-PM-CytKH9/pDNA complexes containing *pDONR-35S-GFP* or *pDONR-Cox2-GFP* reporter constructs for nuclear or mitochondrial expression, respectively. Scale bars represent 20 μm.

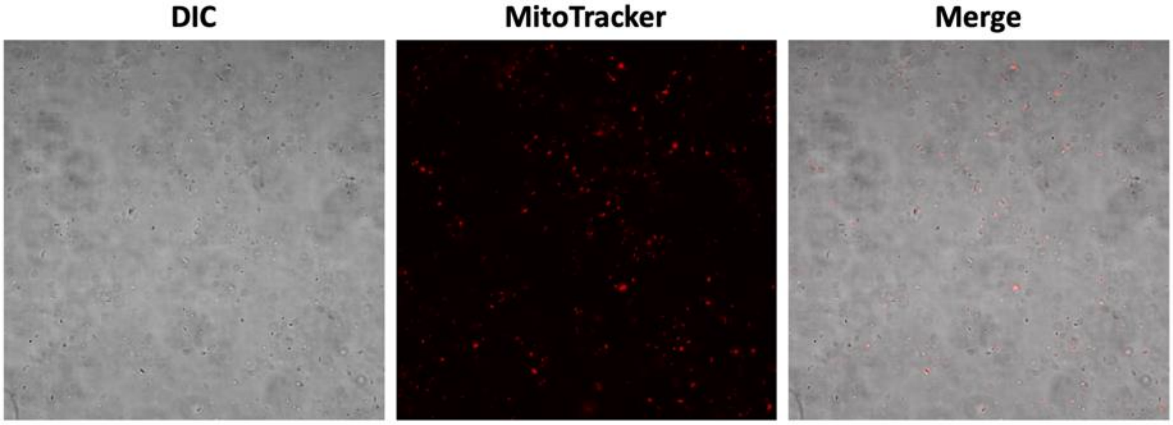

Supplementary Fig. 11. Isolated mitochondria from *A. thaliana* stained with MitoTracker dye.

Isolated mitochondria from *A. thaliana* stained with MitoTracker Red (CMXRos) and visualized by CLSM. Representative CLSM images are shown from 3 isolated mitochondria samples prepared from 12 seedlings each (n=3).

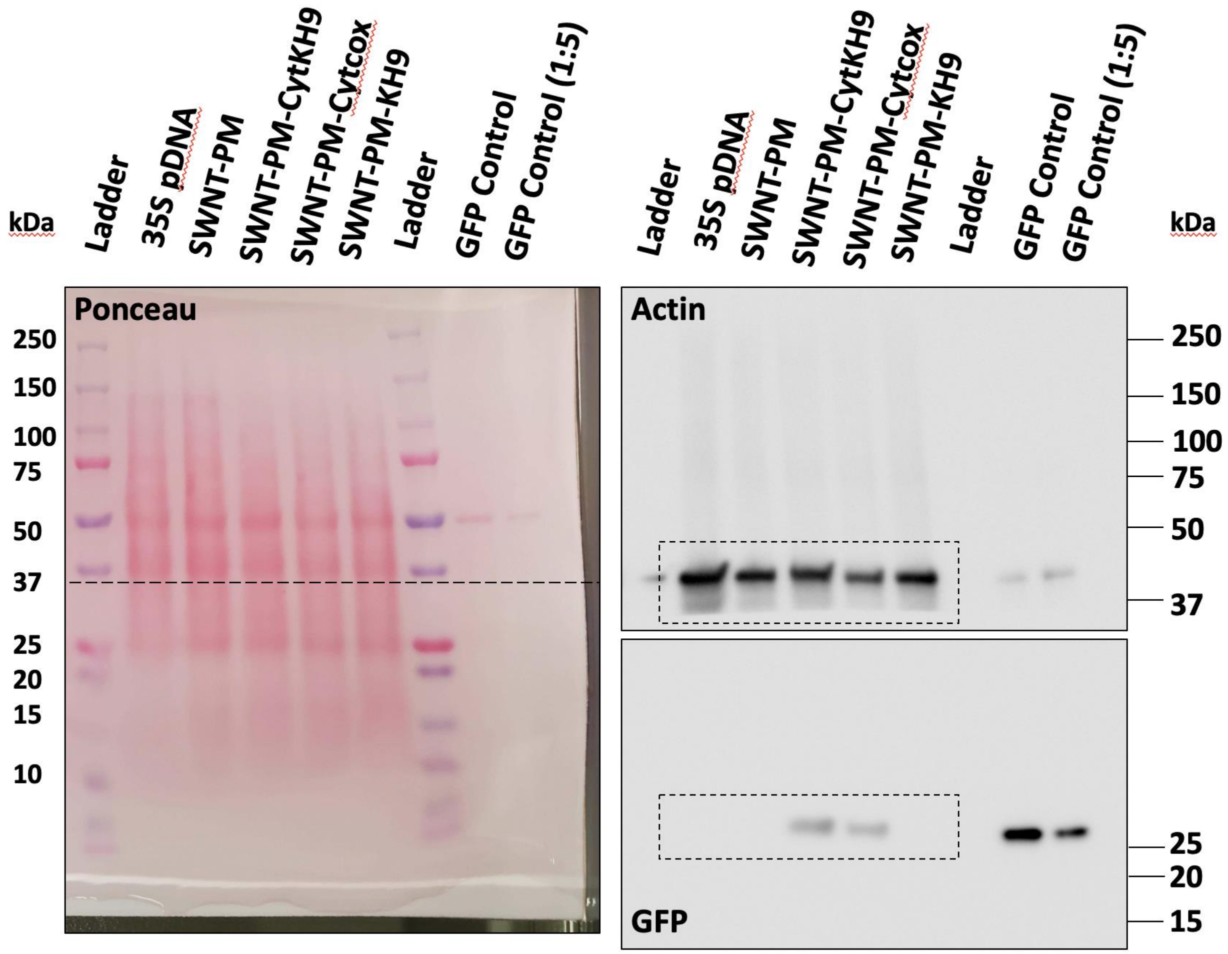

Supplementary Fig. 12. Western blot of GFP expression of cytosolic fraction upon SWNT-PM-Peptide NC infiltration with 35S promoter containing GFP construct.

Uncropped image of representative western blot used in Fig. 5c. Dotted line in the Ponceau stained blot indicates the position where the membrane was cut prior to probing with primary antibody. Dotted lines in the Actin (45 kDa band) and GFP (27 kDa band) blots show the position of bands used in Fig. 5c. GFP was detected using a rabbit polyclonal anti-GFP primary antibody NB600-308 (1:2500; Novus Biologics, USA) and goat anti-rabbit IgG conjugated with horseradish peroxidase (HRP) (1:20000; Abcam, UK). The actin loading control for the cytosolic fraction was probed with a mouse monoclonal anti-actin antibody A0480 (1:2500; Sigma–Aldrich, USA) and goat anti-mouse IgG conjugated with HRP (1:20000; Abcam, UK).

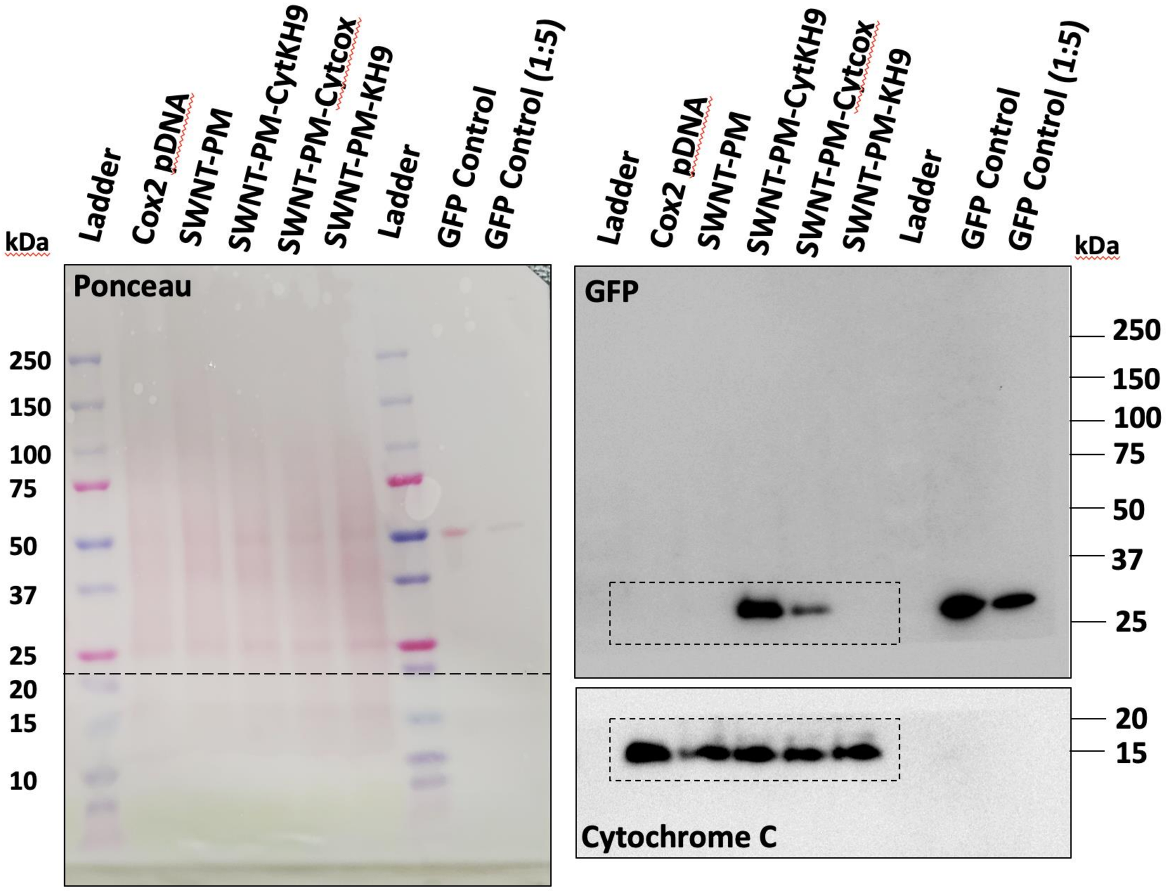

Supplementary Fig. 13. Western blot of GFP expression of mitochondrial fraction upon SWNT-PM-Peptide NC infiltration with Cox2 promoter containing GFP construct. Uncropped image of representative western blot used in Fig. 5d. Dotted line in the Ponceau stained blot indicates the position where the membrane was cut prior to probing with primary antibody. Dotted lines in the Cytochrome C (14 kDa band) and GFP (27 kDa band) blots show the position of bands used in Fig. 5d. GFP was detected using a rabbit polyclonal anti-GFP primary antibody NB600-308 (1:2500; Novus Biologics, USA) and goat anti-rabbit IgG conjugated with horseradish peroxidase (HRP) (1:20000; Abcam, UK). The cytochrome C loading control was probed using a rabbit polyclonal anti-cytochrome C antibody AS08 343A (1:4000; Agrisera, Sweden) and the previously used goat anti-rabbit IgG HRP-conjugated antibody.

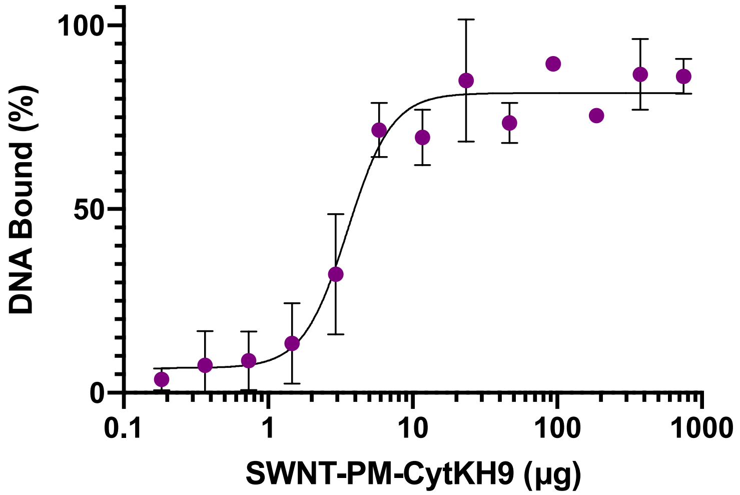

Supplementary Fig. 14. pAtMTTF pDNA complexation with SWNT-PM-CytKH9.

Quantification of gel shift electrophoresis mobility assays showing the amount of residual uncomplexed pAtMTTF pDNA (10 kb) at different SWNT-PM-Peptide NPs/pDNA ratios. Data points represents the mean and the error bars represent the standard deviation of three biological replicates (n=3). Source data are provided as a Source Data file.

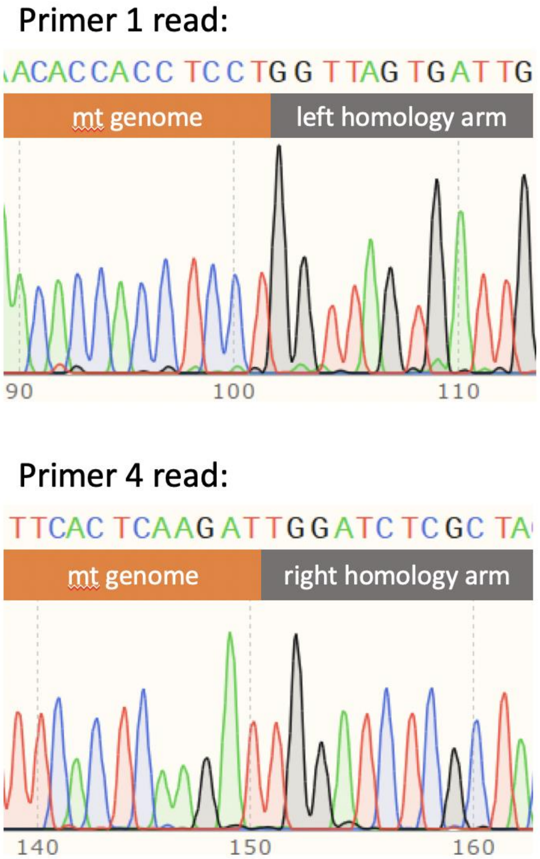

Supplementary Fig. 15. Sequencing of the genotyping PCR products from the 5` and 3` arms of the integrated pAtMTTF1 construct. Sequencing reads spanning the junction between the mitochondrial genome and the homologous sequence from the donor plasmid for recombination on both arms confirmed integration at the expected positions within the mitochondrial genome. Primers 1 and 4 refer to the primers labelled in Fig. 6a. Raw sequence reads can be found in the Supplementary Data 1 and 2.

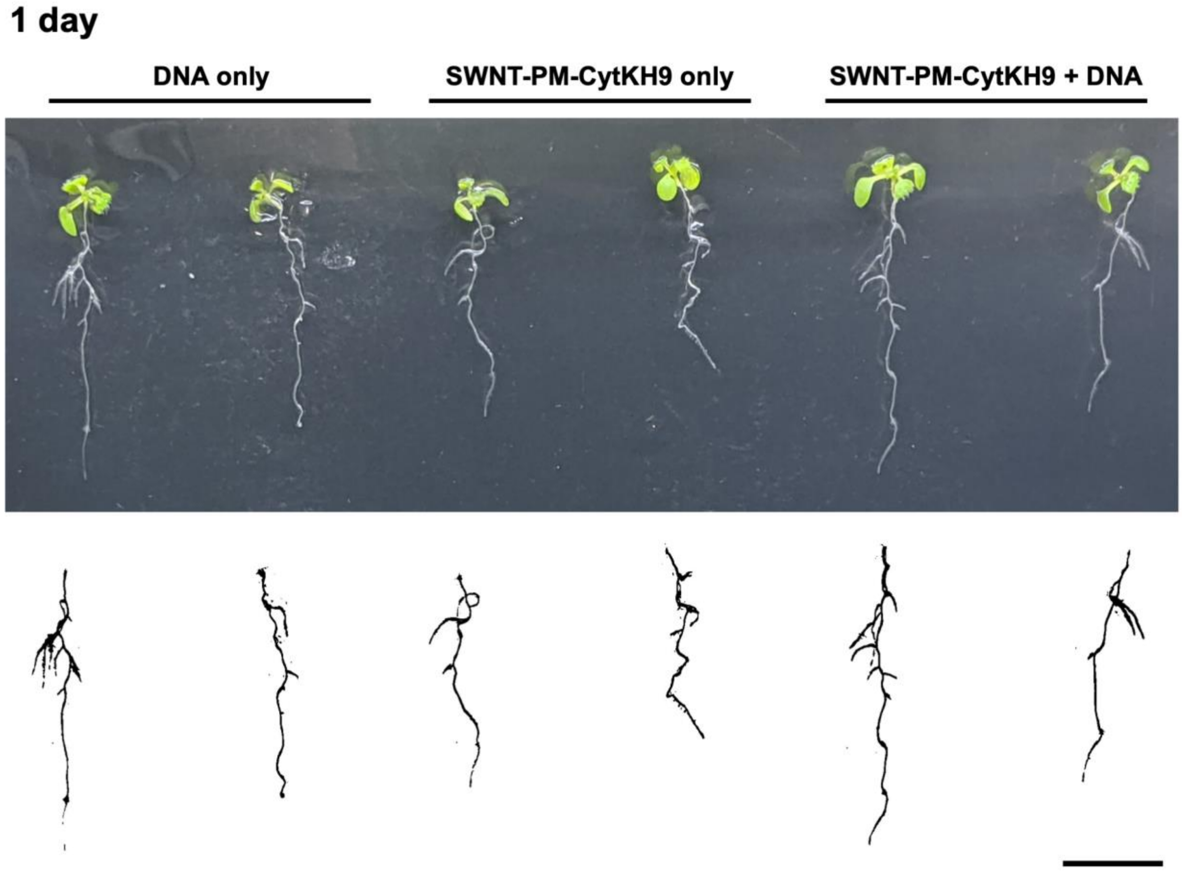

Supplementary Fig. 16. ***A. thaliana*** **root growth 1 day after infiltration with SWNT-PM-CytKH9/pAtMTTF1.**

Area of roots of *A. thaliana* seedlings 1 day after transformation with SWNT-PM-CytKH9/pAtMTTF1 complexes relative to the initial area immediately after transformation. Scale bar indicates 1 cm.

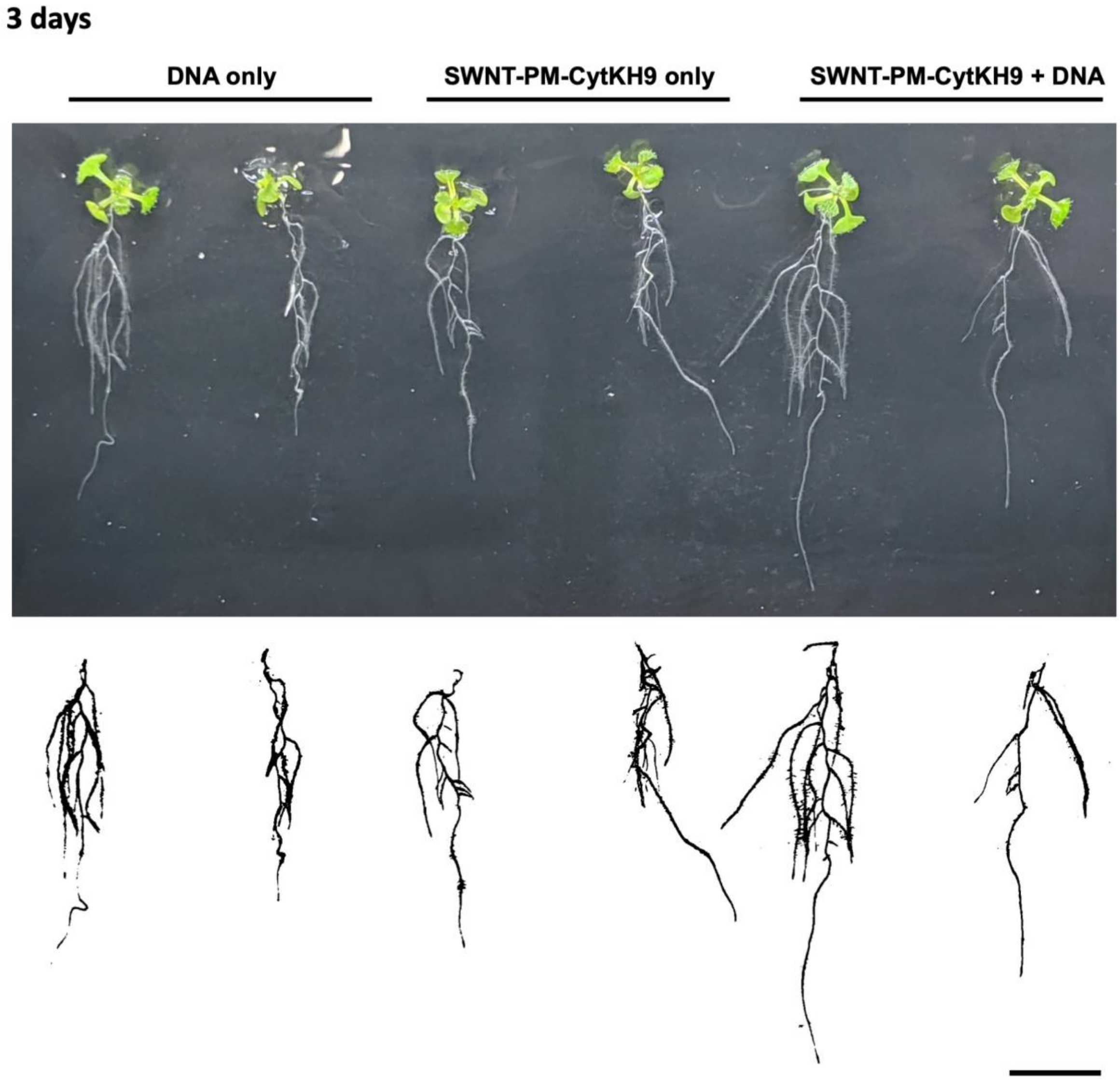

Supplementary Fig. 17. ***A. thaliana*** **root growth 3 days after infiltration with SWNT-PM-CytKH9/pAtMTTF1.**

Area of roots of *A. thaliana* seedlings 3 days after transformation with SWNT-PM-CytKH9/pAtMTTF1 complexes relative to the initial area immediately after transformation. Scale bar indicates 1 cm.

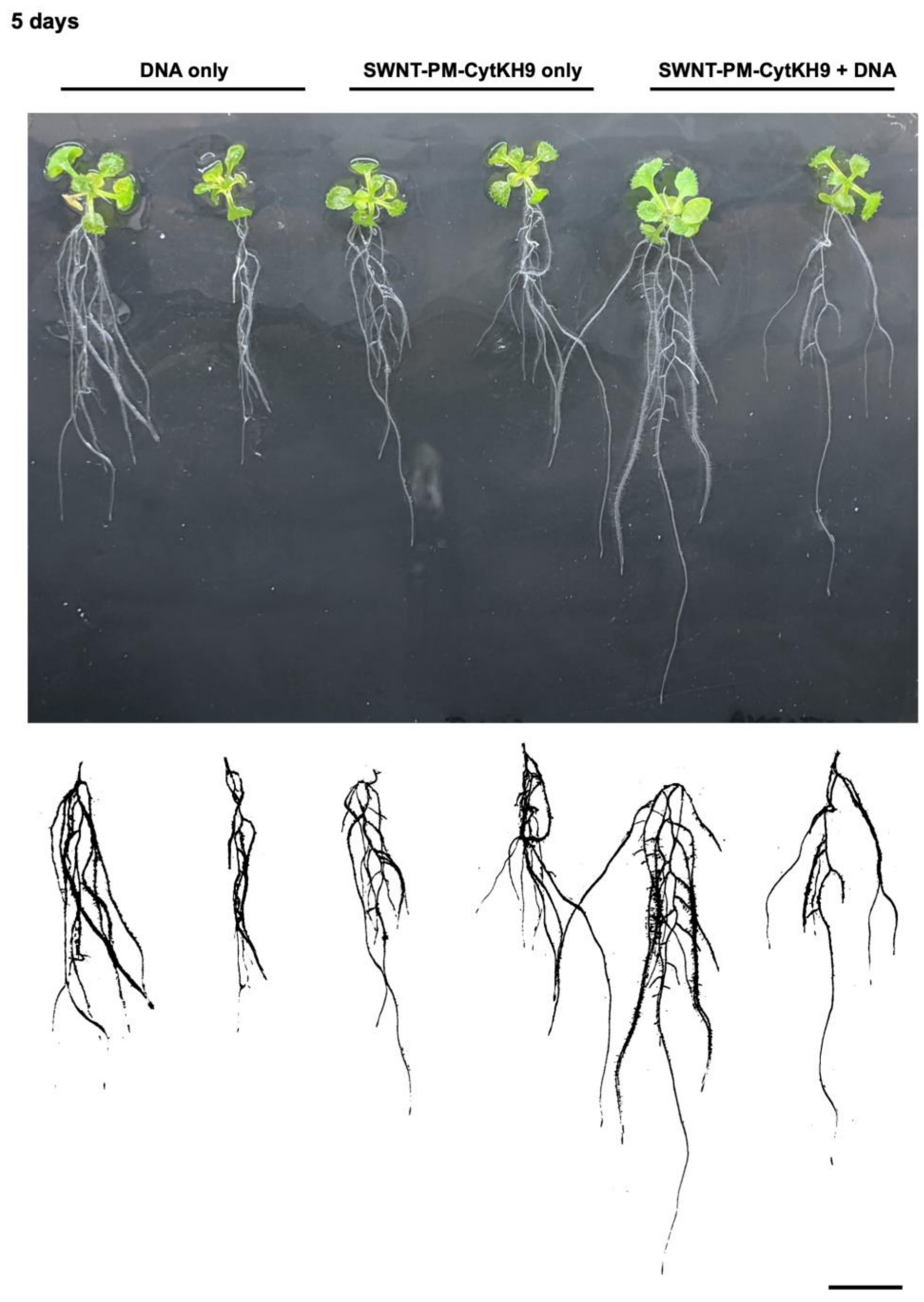

Supplementary Fig. 18. ***A. thaliana*** **root growth 5 days after infiltration with SWNT-PM-CytKH9/pAtMTTF1.**

Area of roots of *A. thaliana* seedlings 5 days after transformation with SWNT-PM-CytKH9/pAtMTTF1 complexes relative to the initial area immediately after transformation. Scale bar indicates 1 cm.

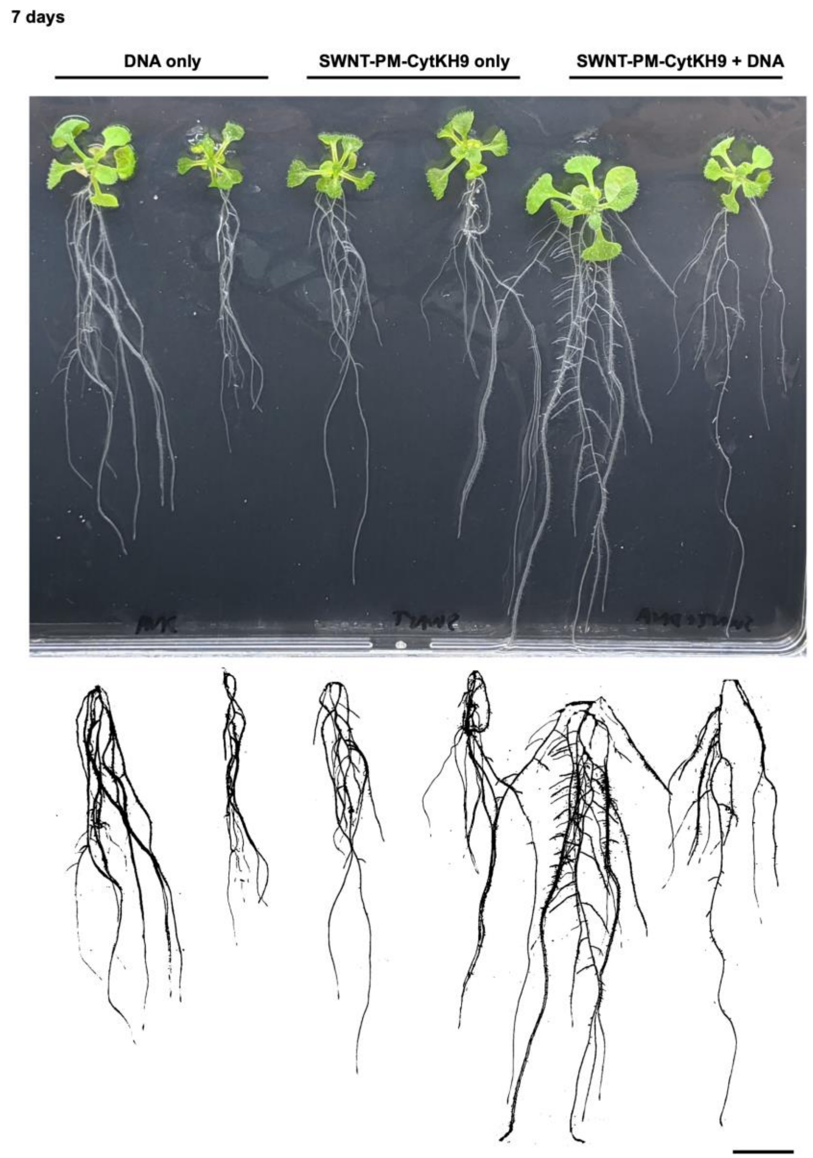

Supplementary Fig. 19. ***A. thaliana*** **root growth 7 days after infiltration with SWNT-PM-CytKH9/pAtMTTF1.**

Area of roots of *A. thaliana* seedlings 7 days after transformation with SWNT-PM-CytKH9/pAtMTTF1 complexes relative to the initial area immediately after transformation. Scale bar indicates 1 cm.

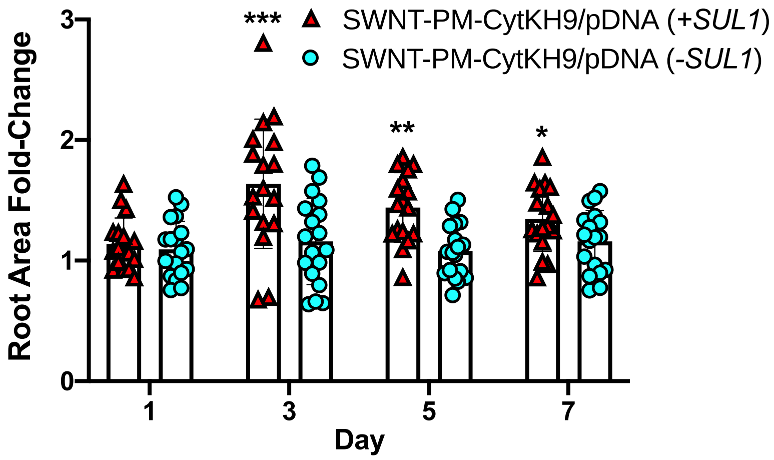

Supplementary Fig. 20. Effect on *A. thaliana* root growth by infiltration of SWNT-PM-CytKH9/pDNA with and without the *SUL1* insert.

A. thaliana root growth after infiltration with SWNT-PM-CytKH9 complexed with pAtMTTF1 pDNA containing the *SUL1* insert (+*SUL1*) and the negative control with the insert removed (-*SUL1*) relative to their respective pDNA only control. Significant differences were observed between the relative root areas (n=18 seedlings) on Day 3, 5, and 7. The bars displayed represent the mean and the error bars represent the standard deviation. Statistical significance between individual samples was determined by two-way ANOVA with matched replicates. *P*-values are 0.0004 for Day 3, 0.0013 for Day 5, and 0.0101 for Day 7. **P*<0.05, ***P*<0.01, ****P*<0.001. Source data are provided as a Source Data file.

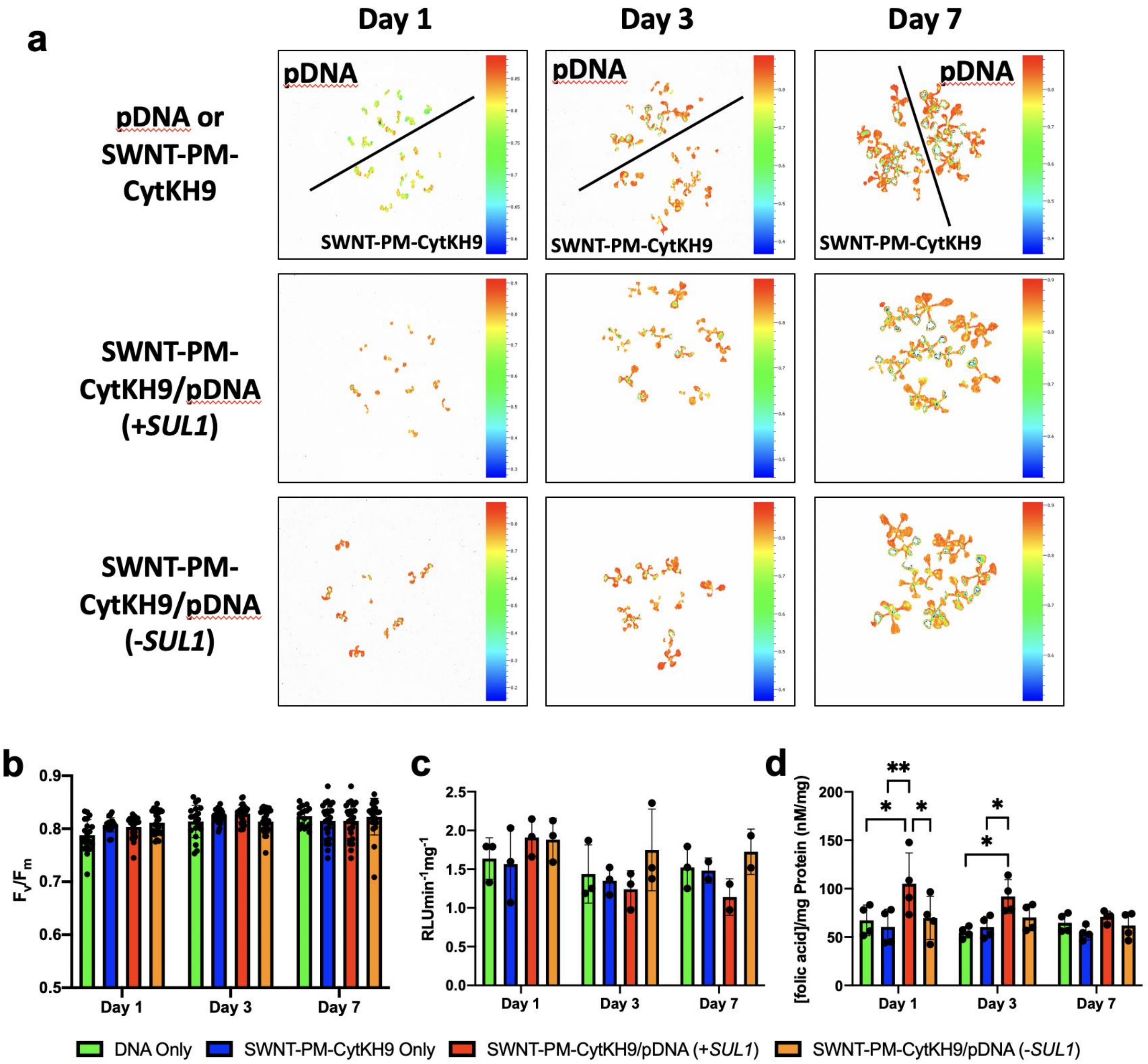

Supplementary Fig. 21. Photosynthetic performance, mitochondrial respiration activities, and intracellular folate concentrations of *A. thaliana* infiltrated with SWNT NCs and its pDNA complex. (a) Representative samples of chlorophyll fluorescence (F_v/F_m) measurements of seedlings at Day 1, 3, and 7 after infiltration with pDNA (+*SUL1*) only, SWNT-PM-CytKH9 with pDNA (+*SUL1* or -*SUL1*) showed no significant differences between the measured samples. **(b)** Quantification of chlorophyll fluorescence (F_v/F_m) of samples from **(a)** across 2 plates per condition containing a minimum of 10 leaf areas (n=20 detected leaf regions per

condition) each using the FluorCam software (Version 7) showed no significant difference between the measured samples. Average values of F_v/F_m ranging from 0.75-0.80 for all sample suggest low levels of stress in plants even after SWNT NC treatment. **(c)** Mitochondrial respiration as quantified by ATP generation from isolated mitochondria from *A. thaliana* showed no significant differences in respiration or mitochondrial stress upon infiltration with SWNT NCs and its complex or DNA only. (n = 3 biological replicates with 12 seedlings per replicate) **(d)** Folic acid quantification of *A. thaliana* infiltrated with SWNT NCs or DNA only show a significant increase in folic acid levels at Days 1 and Day 3 post-infiltration for SWNT-PM-CytKH9/pDNA (+*SUL1*) treated samples relative to the pDNA only and SWNT-PM-CytKH9 samples and at Day 1 relative to SWNT-PM-CytKH9/pDNA (-*SUL1*). (n=4 biological replicates with 12 seedlings per replicate) For all plots, the bars displayed represent the mean and the error bars represent the standard deviation (SD). Statistical significance between individual samples was determined by two-way ANOVA with Tukey's multiple comparisons test. For Day 1, *P*-values are 0.0107 for DNA only and SWNT-PM-CytKH9/pDNA (+*SUL1*), 0.0021 for SWNT-PM-CytKH9 and SWNT-PM-CytKH9/pDNA (+*SUL1*), and 0.0186 for SWNT-PM-CytKH9/pDNA (+*SUL1*) and SWNT-PM-CytKH9/pDNA (-*SUL1*). For Day 3, *P*-values are 0.0131 for DNA only and SWNT-PM-CytKH9/pDNA (+*SUL1*), and 0.0421 for SWNT-PM-CytKH9 and SWNT-PM-CytKH9/pDNA (+*SUL1*). *$P<0.05$, **$P<0.01$, ***$P<0.001$. Source data are provided as a Source Data file.

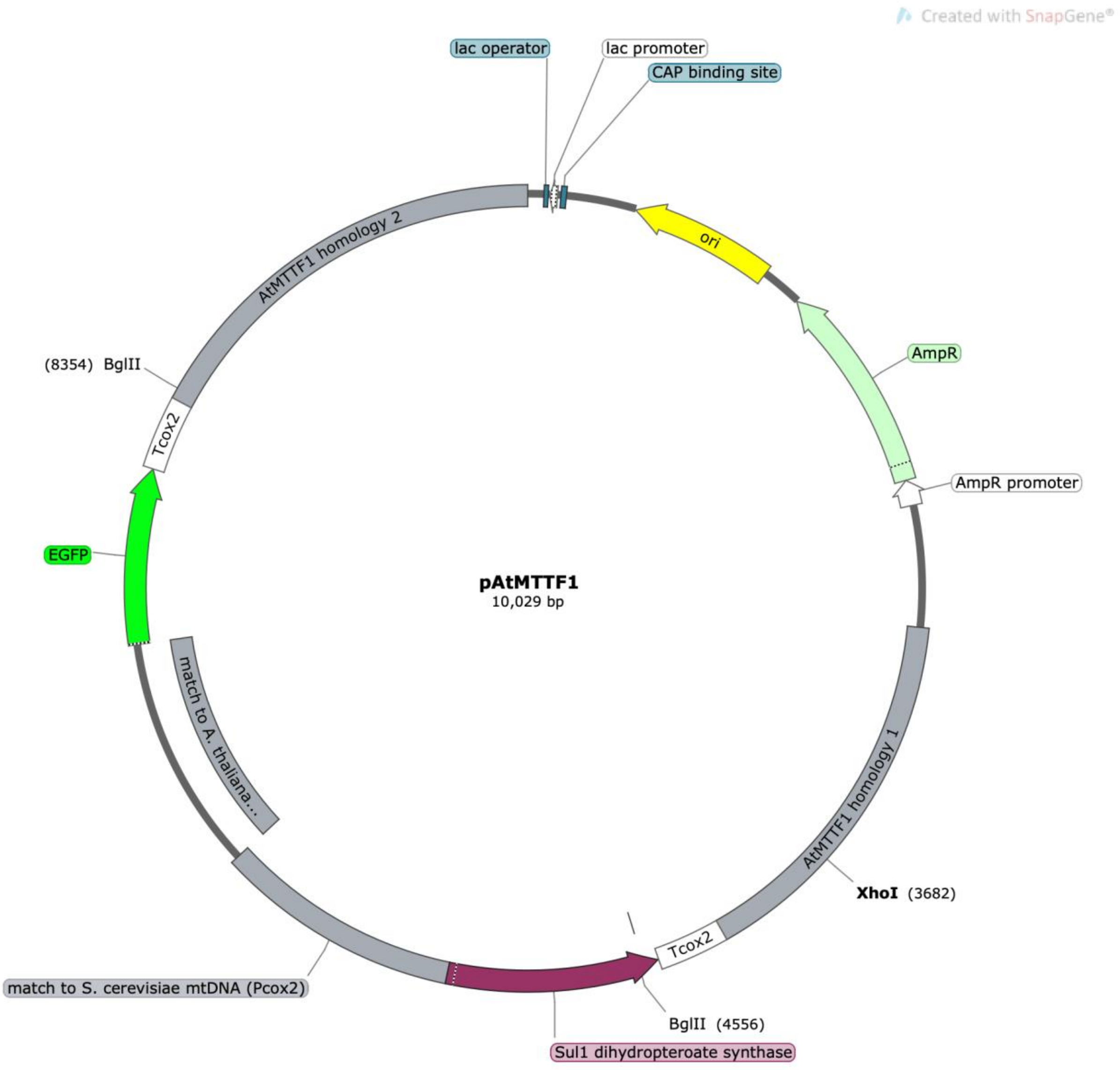

Supplementary Fig. 22. Plasmid map of the reporter construct pAtMTTF1.

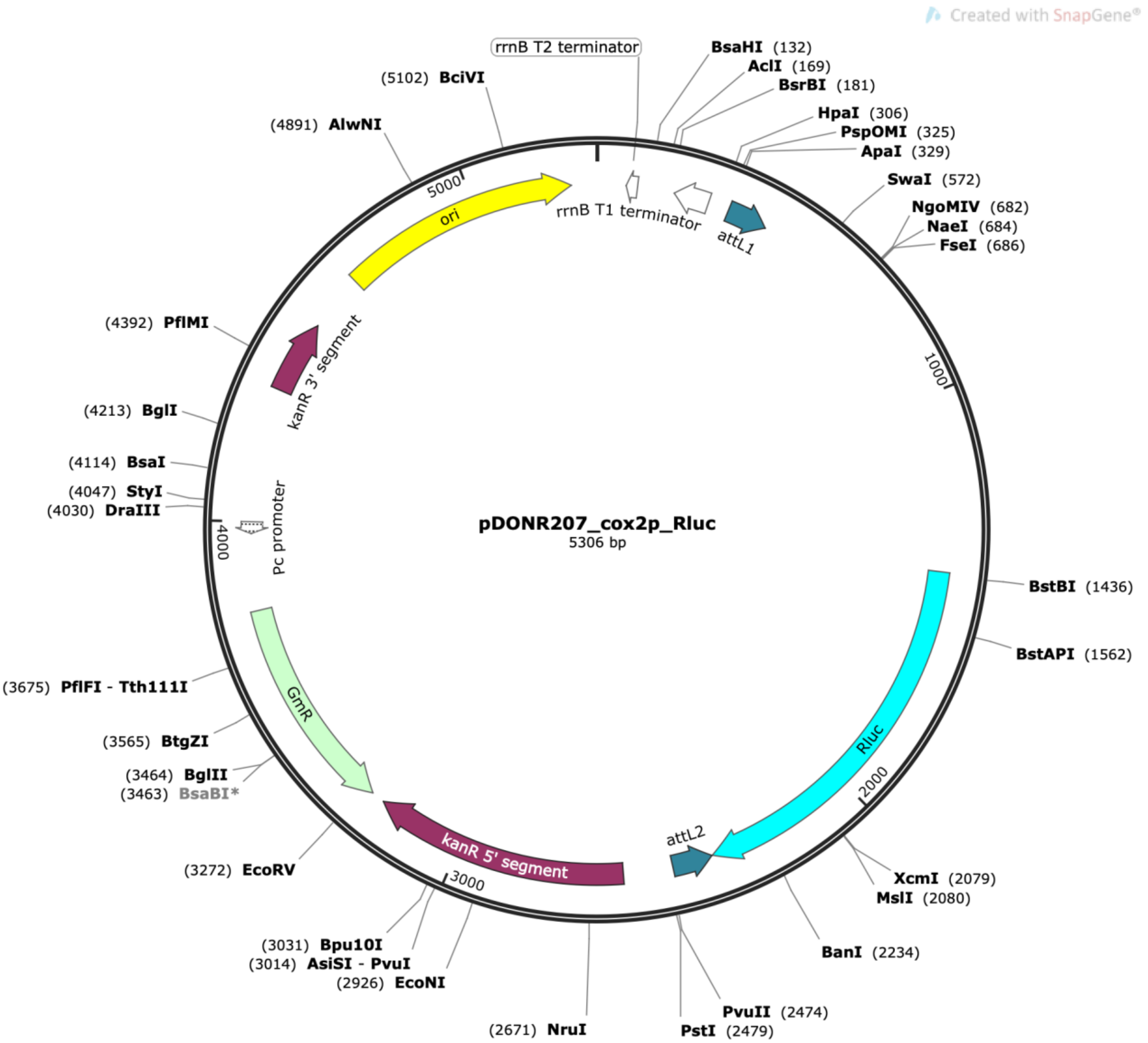

Supplementary Fig. 23. Plasmid map of the reporter construct pDONR-Cox2RLuc.

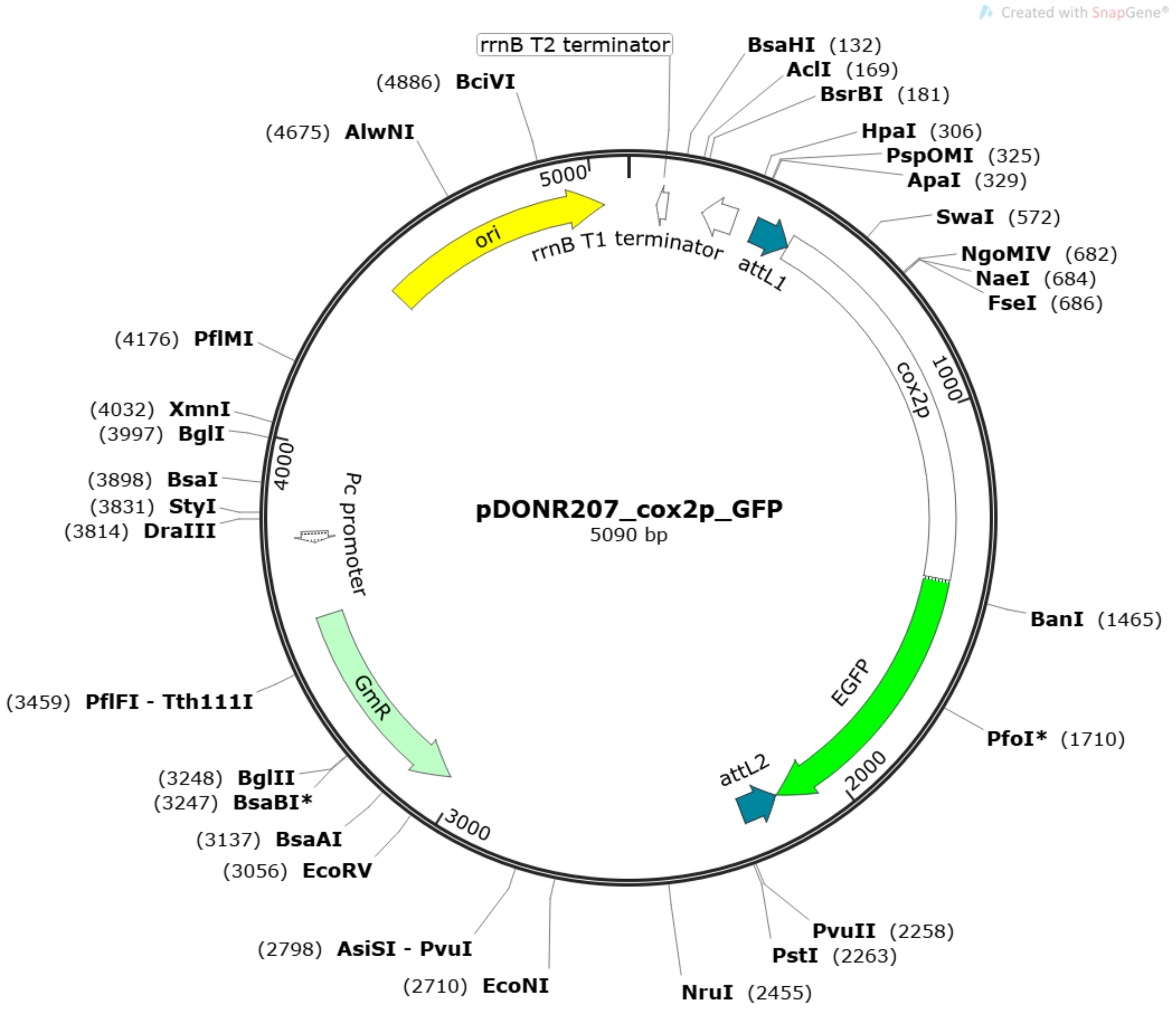

Supplementary Fig. 24. Plasmid map of the reporter construct pDONR207Cox2GFP.

Supplementary Table 1. List of PCR primers used for genotyping experiments in Fig. 6 and Supplementary Fig. 15.

**Name**	**Sequence**	**Primer number (Fig. 6a)**
ATMTTF1-Up	ATCGAGATGCACATGGGATTGG	1
sul-F6	AGCGCAATCACCATCTCGGAAACC	2
GFP-F2	TGGTCCTGCTGGAGTTCGTGAC	3
ATMTTF1-Down	ATGGTTTGCACTTCCGTGTACTAG	4
