# Supplementary material for: Polymer-coated carbon nanotube hybrids with functional peptides for gene delivery into plant mitochondria
Source: Nat Commun. 2022 May 16;13:2417. doi: 10.1038/s41467-022-30185-y (PMC9110379; doi:10.1038/s41467-022-30185-y)
Supplement: Supplementary file 3 — Description of Additional Supplementary Files [file 41467_2022_30185_MOESM3_ESM.pdf]

Description of Additional Supplementary Files

Supplementary Data 1 and 2 contain the raw sequence reads from genotyped PCR products from the 5` and 3` arms of pAtMTTF1 transformants 7 days after infiltration with SWNT-PM-CytKH9/pDNA (Fig. 6 and Supplementary Fig. 15).

Supplementary Data 1. Primer 1 read

Supplementary Data 2. Primer 4 read

Supplementary Data 3 to 5 contain the raw MALDI-TOF/MS analysis of the SWNT NCs Fig. 2d and Supplementary Fig. 4).

Supplementary Data 3. MALDI-TOF/MS analysis of SWNT-PM

Supplementary Data 4. MALDI-TOF/MS analysis of SWNT-PM-Cytcox

Supplementary Data 5. MALDI-TOF/MS analysis of SWNT-PM-CytKH9
