# Supplementary material for: Polymer-coated carbon nanotube hybrids with functional peptides for gene delivery into plant mitochondria
Source: Nat Commun. 2022 May 16;13:2417. doi: 10.1038/s41467-022-30185-y (PMC9110379; doi:10.1038/s41467-022-30185-y)
Supplement: Supplementary file 9 — Reporting Summary [file 41467_2022_30185_MOESM9_ESM.pdf]

nature portfolio

Corresponding author(s): Keiji Numata

Last updated by author(s): Apr 19, 2022

Reporting Summary

Nature Portfolio wishes to improve the reproducibility of the work that we publish. This form provides structure for consistency and transparency in reporting. For further information on Nature Portfolio policies, see our Editorial Policies and the Editorial Policy Checklist.

Statistics

For all statistical analyses, confirm that the following items are present in the figure legend, table legend, main text, or Methods section.

n/a	Confirmed	
☐	☒	The exact sample size (*n*) for each experimental group/condition, given as a discrete number and unit of measurement
☐	☒	A statement on whether measurements were taken from distinct samples or whether the same sample was measured repeatedly
☐	☒	The statistical test(s) used AND whether they are one- or two-sided<br>*Only common tests should be described solely by name; describe more complex techniques in the Methods section.*
☐	☒	A description of all covariates tested
☒	☐	A description of any assumptions or corrections, such as tests of normality and adjustment for multiple comparisons
☐	☒	A full description of the statistical parameters including central tendency (e.g. means) or other basic estimates (e.g. regression coefficient) AND variation (e.g. standard deviation) or associated estimates of uncertainty (e.g. confidence intervals)
☐	☒	For null hypothesis testing, the test statistic (e.g. *F*, *t*, *r*) with confidence intervals, effect sizes, degrees of freedom and *P* value noted<br>*Give P values as exact values whenever suitable.*
☒	☐	For Bayesian analysis, information on the choice of priors and Markov chain Monte Carlo settings
☒	☐	For hierarchical and complex designs, identification of the appropriate level for tests and full reporting of outcomes
☒	☐	Estimates of effect sizes (e.g. Cohen's *d*, Pearson's *r*), indicating how they were calculated

Our web collection on statistics for biologists contains articles on many of the points above.

Software and code

Policy information about availability of computer code

Data collection	Raman microscopy: JASCO Spectra Manager (Version 2.13)<br>Confocal scanning microscopy: Carl Zeiss Zen (Version 12.0)<br>Field emission scanning electron microscopy: SmartSEM (Version 6.01) and ZeissAtlas (Version 5.1.4.27)<br>Molecular dynamics simulations: AMBER18<br>Quantum mechanics calculations: Gaussian16<br>Chlorophyll Fluorescence measurements: FluorCam (Version 7)
Data analysis	Scanning atomic force microscopy: Gwyddion (Version 2.58)<br>Image analysis and quantification: ImageJ Fiji (Version 2.1)<br>Statistical analysis: Graphpad Prism 8.0<br>Field emission scanning electron microscopy: SmartSEM (Version 6.01) and ZeissAtlas (Version 5.1.4.27)<br>Molecular dynamics simulation: AMBER18, PyMol Version 1.8.5<br>Chlorophyll Fluorescence measurements: FluorCam (Version 7)

For manuscripts utilizing custom algorithms or software that are central to the research but not yet described in published literature, software must be made available to editors and reviewers. We strongly encourage code deposition in a community repository (e.g. GitHub). See the Nature Portfolio guidelines for submitting code & software for further information.

Data

Policy information about availability of data

All manuscripts must include a data availability statement. This statement should provide the following information, where applicable:

- Accession codes, unique identifiers, or web links for publicly available datasets
- A description of any restrictions on data availability
- For clinical datasets or third party data, please ensure that the statement adheres to our policy

The reporting summary for this article is included in the Supplementary Information. All data supporting the findings of this study are available within the article, the Supplementary Information or in the Source data file. The data underlying all graphs and plots in the figures and supplementary information are provided within the Source Data file. Mass spectrometry data and PCR sequencing data are included as Supplementary Data. Source data are provided as a Source data file. Any other further information can be provided by the corresponding author upon reasonable request.

Field-specific reporting

Please select the one below that is the best fit for your research. If you are not sure, read the appropriate sections before making your selection.

☒ Life sciences ☐ Behavioural & social sciences ☐ Ecological, evolutionary & environmental sciences

For a reference copy of the document with all sections, see nature.com/documents/nr-reporting-summary-flat.pdf

Life sciences study design

All studies must disclose on these points even when the disclosure is negative.

Sample size	No statistical analysis was used to determine sample sizes. Sample sizes were determined as reasonable and appropriate for each experiment based on literature and observed sample variance in each individual experiment. Individual sample bias were minimized by averaging large sample sizes with a large number of seedlings and the use of internal controls where possible.<br><br>SEM micrographs are shown from 4 separate imaged drops (n=4) from each prepared sample condition. Each sample was imaged multiple times over a large sample area and representative images are shown.<br>MS data were collected with a minimum of 6 spectra for each sample.<br>Zeta potentials for 3 sample preparations are shown.<br>AFM heights were taken from 3 separately prepared drops on the mica substrate and imaged in a minimum of 3 overall locations. A minimum of 25 height profiles from individually discernible SWNTs were taken and used for analysis.<br>Raman intensities were taken as an average of 64 spectra taken over the mapped area (>200 um^2) from mitochondria isolated from 12 seedlings that were imaged in more than 3 areas per sample.<br>Luminescence values were taken from 6 separate experiments with 3 technical replicates with 12 individual seedlings per experiment as shown in the graph.<br>Cell viability were performed similarly with 6 biological replicates.<br>Confocal microscopy images are shown from a minimum of 5 representative biological replicates that were imaged in multiple areas (>5) per seedling.<br>Western blot analysis was performed with 5 separate experiments, with each sample containing a minimum of 60 seedlings that were used for mitochondrial and cytosolic fraction isolation.<br>Genotyping PCR was performed on a minimum of 30 biological replicates and 6 to 8 representative samples are shown in Fig. 6.<br>Root growth quantification was performed on 10 to 12 biological replicates of individual seedling that were imaged at the displayed time points separately. Images from 5 representative seedlings are shown in Figs. S16-19.<br>DNA complexation experiments were performed using 3 separately prepared samples.<br>Chlorophyll fluorescence experiments are shown with a minimum of 20 leaf areas across 12 seedlings on 2 plates, each measured 2 times.<br>Mitochondrial respiration were measured using mitochondria isolated from 12 seedlings with 3 biological replicates per sample condition.<br>Folic acid quantification was measured 4 times from 12 seedlings per treatment condition per replicate.
Data exclusions	No data were excluded from analysis.
Replication	In Fig. 2, SEM micrographs are shown from 4 imaged drops for each sample condition. Polymer analysis was performed on MS data as plot in Fig S4. Representative spectra are shown from a minimum of 10 spectra collected from each MALDI sample, which showed similar results in each. MS data are provided in Supplementary Information. Zeta potentials are shown for three biological replicates that all showed similar values. AFM heights were determined from 25 height profiles taken from 3 biological replicates as shown in the plot.<br>In Fig. 3 and S8, normalized Raman intensities were taken as the average of an 64 spectra taken. Representative Raman mapping images was taken from isolated mitochondria from a minimum of 12 seedlings. A minimum of 3 areas were mapped containing a minimum of 500 pixels each were shown and reflect the results shown. Confocal microscopy micrographs are shown from a minimum of 5 representative biological replicates.<br>In Fig. 4, luminescence values were taken from 6 biological replicates containing 3 technical replicates per data point as shown in the graph. Cell viability was calculated from 6 biological replicates.<br>In Fig. 5 and S10, confocal microscopy micrographs are shown from a minimum of 5 representative biological replicates. Western blot quantification was performed with 5 biological replicates, each containing 60-65 seedlings per sample.<br>In Fig. 6, genotyping PCR was performed on 6 to 8 biological replicates as shown in the agarose gel that reflect a minimum of 30 biological

replicates performed, and root growth quantification was performed on 10 to 12 biological replicates.
In Fig. S14, the mean and SD are plotted from 3 individually prepared samples.
In Fig. S20, normalized root lengths are shown for 18 individually tracked seedlings.
In Fig. S21, chlorophyll fluorescence (Fv/Fm) are shown for a minimum of 20 leaf areas detected by Fluorocam (Version 7) across a minimum of 12 seedlings on 2 plates that were measured 2 times. Mitochondrial respiration were measured 3 times from 12 seedlings per treatment condition per replicate. Folic acid quantification was measured 4 times from 12 seedlings per treatment condition per replicate.

Randomization

Sampling for individual experiments was performed using randomly selected plants that were grown together.
Computational analysis were applied equally and used for quantification experiments.
Analysis was performed on known samples that could be visually confirmed.
Areas sampled for image analysis was determined randomly and were imaged over large areas to minimize bias.

Blinding

Blinding was not relevant to this study due to the overall study design. Analysis was performed on known samples that could be visually confirmed.
Overall bias was minimized by increasing overall replicate size with large sample numbers for the characterization of the SWNTs.
The use of computational analysis that were applied equally for gene expression and genotyping experiments as well for growth-related experiments was used to minimize inherent experimenter bias.

Reporting for specific materials, systems and methods

We require information from authors about some types of materials, experimental systems and methods used in many studies. Here, indicate whether each material, system or method listed is relevant to your study. If you are not sure if a list item applies to your research, read the appropriate section before selecting a response.

Materials & experimental systems

n/a	Involved in the study
☐	☒ Antibodies
☒	☐ Eukaryotic cell lines
☒	☐ Palaeontology and archaeology
☒	☐ Animals and other organisms
☒	☐ Human research participants
☒	☐ Clinical data
☒	☐ Dual use research of concern

Methods

n/a	Involved in the study
☒	☐ ChIP-seq
☒	☐ Flow cytometry
☒	☐ MRI-based neuroimaging

Antibodies

Antibodies used

Anti-GFP Antibody - Novus Biologicals (NB600-308) (1:2500)
Anti Plant Cytochrome c Antibody - Agrisera (AS08 343A) (1:4000)
Anti Plant Actin Antibody - Sigma Aldrich (A0480) (1:4000)
Goat Anti-Mouse IgG H&L (HRP) (ab6789) (abcam) (1:20000)
Goat Anti-Rabbit IgG H&L (HRP) (ab6721) (abcam) (1:20000)

Validation

Anti-GFP Antibody - Novus Biologicals (NB600-308) Assay by immunoelectrophoresis resulted in a single precipitin arc against anti-Rabbit Serum and purified and partially purified Green Fluorescent Protein (Aequorea victoria). Tested for Western blot analysis. Complete validation statements are available on the manufacturer's website (Lot 45455).

Anti Plant Cytochrome c Antibody - Agrisera (AS08 343A) Confirmed reactivity for Cytochrome c, located in inner mitochondrial membrane, for Arabidopsis thaliana, Brassica oleracea, Glycine max, Pisum sativum, Zea mays. Tested for Western blot analysis. Complete Validation statements are available on the manufacturer's website (Lot 1208).

Anti Plant Actin Antibody - Sigma Aldrich (A0480) Monoclonal Anti-Actin (plant) antibody recognizes actin in several plant species including Dictyostelium. In Arabidopsis, the antibody recognizes all eight actins isoforms (ACT1, 2, 3, 4, 7, 8, 11 and 12). Tested for Western blot analysis. Complete validation statements are available on the manufacturer's website (Lot 059M4799V).

Goat Anti-Mouse IgG H&L (HRP) (ab6789) (abcam) This Ab was prepared from monospecific antiserum by immunoaffinity chromatography with the entire mouse IgG as the immunogen. HRP is conjugated for visualization. Tested for Western blot analysis. Complete validation statements are available on the manufacturer's website (Lot GR3357864-1).

Goat Anti-Rabbit IgG H&L (HRP) (ab6721) (abcam) This Ab was prepared from monospecific antiserum by immunoaffinity chromatography with the entire rabbit IgG as the immunogen. HRP is conjugated for visualization. Tested for Western blot analysis. Complete Validation statements are available on the manufacturer's website (Lot 1304).
